# Increased rCBV in status epilepticus

**DOI:** 10.1007/s00415-012-6443-4

**Published:** 2012-02-14

**Authors:** J. J. G. Rath, M. Smits, F. Ducray, M. J. van den Bent

**Affiliations:** 1 Department of Neurology, Haga Teaching Hospital, The Hague, The Netherlands; 2 Department of Radiology, Erasmus Medical Center, Rotterdam, The Netherlands; 3 Department of Neuro-oncology, Hôpital Neurologique, Lyon, France; 4 Department of Neuro-oncology, Neurology, Daniel den Hoed Cancer Center, Erasmus Medical Center, Groene Hilledijk 301, 3075 EA Rotterdam, The Netherlands

Dear Sirs,

The estimated relative cerebral blood volume (rCBV) is a semiquantitative parameter that correlates with tissue vascularisation. High-grade gliomas are characterised by an increased macrovasculature and microvasculature, and therefore associated with an increased rCBV [1]. In neuro-oncology, high rCBV is used to distinguish tumoral from non-tumoral increase in lesion size, e.g, pseudoprogression and radiation necrosis from real tumour progression. However, in high-grade glioma an increase in rCBV without tumour progression can also be observed in (non-convulsive) status epilepticus, as we will describe below.

**Fig. 1:**
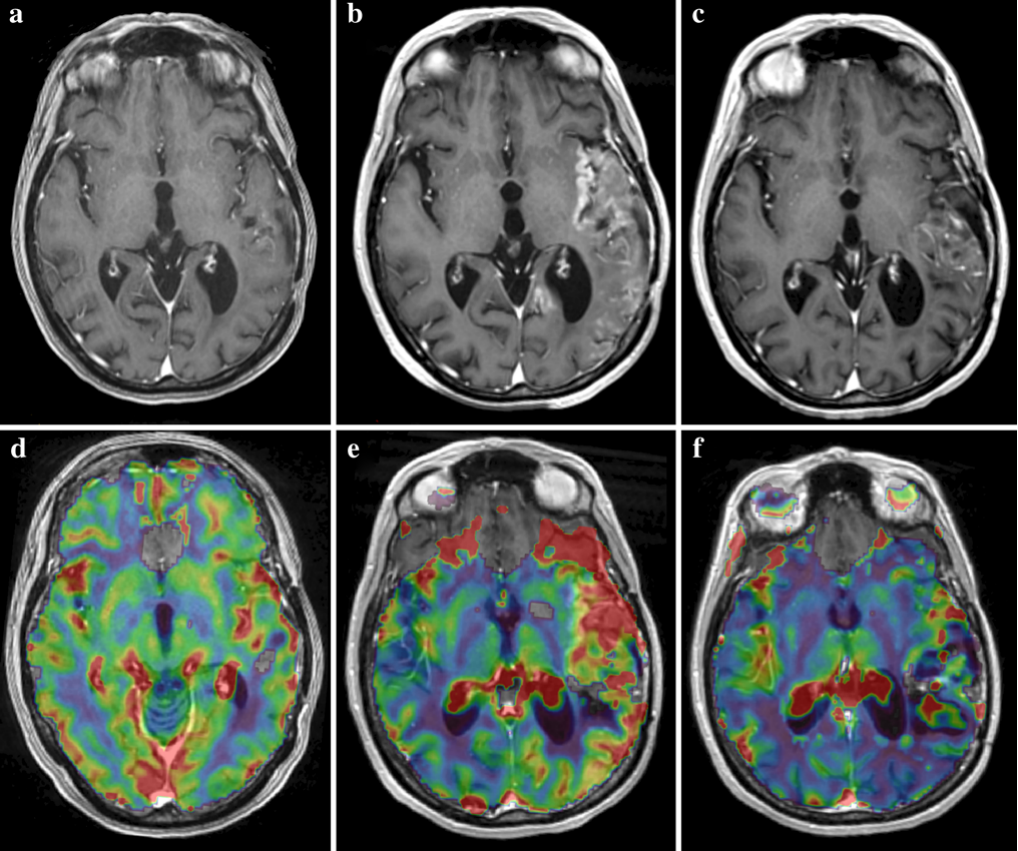
T1-weighted MRI with gadolinium **a** 5 months prior to admission, **b** during admission, **c** 2 months after admission, **d**–**f** DSC perfusion-weighted MRI **d** 5 months prior to admission, **e** during admission, and **f** 2 months after admission

A 45-year-old woman was admitted to our hospital because of a complete paresis of the right arm and motor speech impairment. Thirteen years before she had been treated for an anaplastic oligodendroglioma of the left temporal lobe with surgery followed by radiotherapy (59.4 Gy) and chemotherapy (6 cycles of procarbazine, CCNU, vincristin). Every 6 months a magnetic resonance imaging (MRI) scan of the brain was performed showing a post-surgery cyst and local subtle enhancement, which remained stable over this period (Fig. 1a). Dynamic susceptibility contrast (DSC) perfusion-weighted MR imaging [T2*w gradient echo echo-planar imaging with repetition time of 2,000 ms; 60 phases; contrast bolus injection after 10 phases at 5 ml/s of 12 ml of Gadovist^®^ 1.0 (Bayer Schering Pharma) followed by 20 ml 0.9% NaCl; preload bolus of 3 ml of Gadovist^®^ 1.0 (Bayer Schering Pharma) 5 min prior to DSC perfusion imaging] performed 5 months before admission showed rCBV ratios of 1.09 (insular L) and 1.18 (occipital L) (Fig. 1d). One week before admission, she had woken up with a headache, a paresis of her right arm and an increased speech impairment. In the following days her headache subsided and her paresis was fluctuating. On admission she showed a mixed aphasia, right hemianopia and a complete paresis of her right arm. An electroencephalogram (EEG) showed clear epileptic discharges located left centroparietal without clinical corresponding symptoms. A MRI scan of the brain showed cortical swelling with extensive cortical enhancement of the left temporal lobe (Fig. 1b), and perfusion-weighted imaging showed rCBV ratios of 2.24 (insular L) and 1.64 (occipital L) (Fig. 1e), suggesting tumour progression. The patient was treated with anti-epileptic drugs followed by an improvement of clinical symptoms. A repeat MRI made 2 months later showed a decrease of enhancement (Fig. 1c) and of rCBV ratios (1.07 insular L and 0.78 occipital L) (Fig. 1f) to near-baseline values, indicating the increase in contrast enhancement and rCBV was not due to tumour progression.

Radiological seizure-associated abnormalities in epilepsy have been described before. The changes that can be seen on MR imaging are mainly characterised by focal cortical swelling and diffusion restriction due to cytogenic and vasogenic oedema [2], but leptomeningeal and/or cortical enhancement can also be seen [3], suggesting a disruption of the blood brain barrier. Recently, Rheims et al. [4] described ten patients with extensive transient cortical and leptomeningeal contrast-enhancement in the context of seizures in brain tumour patients previously treated with radiotherapy. They argue that the marked and extensive enhancement they observed (labelled peri-ictal pseudoprogression), might be related to a postradiation vasculopathy. Although they also mention a moderate increase of rCBV in two patients, no data on this are provided, and follow-up rCBV is not mentioned. It is therefore unsure whether their findings were also transient.

Another late reversible complication seen in brain tumour patients after radiation therapy is the so-called SMART syndrome (stroke-like migraine attacks after radiation therapy) [5]. To what extent our case is part of the SMART syndrome and to what extent the SMART syndrome is due to seizures are at present unclear.

Our case shows that, despite the known association between increased rCBV and tumour progression, rCBV increase should be interpreted with caution, and peri-ictal pseudoprogression should be considered as well.
